# Effect of Oral Administration of Collagen Peptide OG-5 on Advanced Atherosclerosis Development in ApoE^−/−^ Mice

**DOI:** 10.3390/nu16213752

**Published:** 2024-10-31

**Authors:** Yijie Yang, Bo Li

**Affiliations:** 1 Department of Nutrition and Health, China Agricultural University, Beijing 100193, China; 2 College of Food Science and Nutritional Engineering, China Agricultural University, Beijing 100083, China; 3 Key Laboratory of Functional Dairy, Ministry of Education, Beijing 100083, China

**Keywords:** atherosclerosis, vascular smooth muscle cells, collagen peptide

## Abstract

Background/Objectives: Atherosclerosis is a chronic inflammatory disease of the arterial wall, which involves multiple cell types. Peptide OG-5 is identified from collagen hydrolysates derived from *Salmo salar* and exhibits an inhibitory effect on early atherosclerosis. The primary objective of this study was to investigate the impact of OG-5 on advanced atherosclerotic lesions as well as its stability during absorption. Methods: In this study, the ApoE-/- mice were employed to establish advanced atherosclerosis model to investigate the treatment effect of peptide OG-5. Results: The results showed that oral administration of OG-5 at a dosage of 150 mg/kg bw resulted in a 30% reduction in the aortic plaque formation area in ApoE^−/−^ mice with few bleeding risks. Specifically, intervention with a low dose of OG-5 (50 mg/kg bw), initiated in the early stage of atherosclerosis, continues to provide benefits into the middle and late stages without bleeding risks. Furthermore, treatment of OG-5 increased expression levels of contractile phenotype markers and reduced the accumulation of lipoprotein in VSMCs induced by ox-LDL. Peptide OG-5 could ensure transport across Caco-2 cell monolayers, exhibiting a P_app_ value of 1.80 × 10^−5^ cm/s, and exhibited a robust stability in plasma with remaining content >70% after 8 h incubation. In vivo studies revealed that OG-5 reached maximum concentration in blood after 120 min. Conclusion: The present results demonstrate the potential efficacy of peptide OG-5 as a promising agent for intervention in anti-atherogenesis strategies.

## 1. Introduction

Atherosclerosis (AS) is a chronic and persistent inflammatory disease of the arterial wall, which serves as the underlying pathological mechanism for a wide array of prevalent cardiovascular diseases (CVDs). The intricate pathological progression of AS encompasses a myriad of cellular events, which include sustained platelet activation and subsequent granule secretion, the accumulation of macrophages and vascular smooth muscle cells (VSMCs), alongside the infiltration and morphological alterations of diverse immune cells [1]. In the early stage of lesion initiation, classic monocytes enter the intima induced by chemokines and mature into macrophages. Additionally, VSMCs in the tunica media undergo a phenotype switch from contractile to synthetic dedifferentiation under the stimulation of cytokines. The synthetic VSMCs obtain proliferation and migration ability to migrate into the intima. These cells express scavenger receptors, enabling them to bind lipoprotein particles and subsequently transform into foam cells, thereby accelerating the development and progression of atherosclerotic plaque formation [2,3]. Platelets also serve as pivotal mediators of inflammation and exhibit immunomodulatory activities, which significantly contribute to the initiation and progression of AS [4]. Upon platelet activation, the release of granules initiates coagulation cascades and facilitates the recruitment of additional cellular components to the site of vascular injury during inflammatory responses. Furthermore, platelets exert a regulatory effect on cholesterol metabolism through their ability to bind and modify low-density lipoprotein (LDL) particles. This process significantly contributes to the generation of lipid-laden cells, which are pivotal in the development and progression of atherosclerotic plaques [5].

However, traditional drugs in the treatment of AS, such as statins and aspirin, are subject to limitations owing to their associated side effects. A clinical study has demonstrated that administering low-dose aspirin as a primary prevention strategy in elderly adults is associated with a notably elevated risk of major hemorrhagic events and fails to confer a statistically significant reduction in the risk of cardiovascular diseases (CVDs) compared to placebo intervention [6]. It has been observed in clinical trials that daily aspirin prophylaxis among healthy elderly individuals leads to an increased risk of all-cause mortality compared to placebo prevention [7]. Additionally, more than 50% of patients did not benefit from the intervention of statins after receiving intensive lipid-lowering drugs. Instead, they had side effects such as diabetes and muscle complications [8]. Thus, it is essential for the development of a novel compound that can directly restrain atherogenesis, especially via inhibition against foam cell formation.

Collagen, characterized by its unique structural feature of repeating amino acid motifs, serves as an abundant natural source of bioactive peptides. Specifically, within the Gly-X-Y sequence, proline and hydroxyproline frequently occupy the X and Y positions, respectively. Collagen hydrolysates have been reported to involve multiple bioactivities, especially in the treatment of osteoarthritis, which is robustly substantiated through both animal model experimentation and molecular modeling of intermolecular interactions [9]. The clinical manifestations of naturally occurring osteoarthritis in dogs were not only halted but also exhibited improvement following a 12-week period of oral supplementation with bone collagen peptides [10]. In our previous research, we isolated a range of Hyp-Gly (OG)-containing peptides from collagen hydrolysates (CHs) in *Salmo salar* and found potent in vitro antiplatelet activity. Among these peptides, OGEFG (OG-5) emerged as a particularly effective inhibitor of thrombus formation, as demonstrated in both Fecl_3_-induced arterial thrombosis and carrageenan-induced mouse tail thrombosis models. Notably, OGEFG achieved this effect without significantly increasing the risk of bleeding, which it accomplished by targeting the P2Y12 receptor on platelets [11,12]. Emerging evidence suggests that the P2Y12 receptor holds a dual role in cardiovascular health, not only serving as a well-recognized target for anti-thrombotic therapies but also potentially mediating atherogenic processes within the vessel wall. This latter function appears to be independent of platelet activation, as the receptor is widely distributed across endothelial cells, VSMCs, and platelets, all of which contribute to the development of AS [13,14,15]. Furthermore, a recent study has reported that activation of the P2Y12 receptor results in reduced cholesterol efflux and facilitates the formation of foam cells derived from VSMCs in the context of advanced AS [16]. Therefore, the P_2_Y_12_ receptor emerges as a promising target for the development of inhibitors aimed at modulating foam cell differentiation through direct regulation of cholesterol efflux. Fortunately, our previous study has shown that treatment of OG-5 could prevent the formation of early AS in ApoE^−/−^ mice caused by a fat diet [12], indicating its potential for the primary prevention of AS. However, whether peptide OG-5 had a therapeutic effect on AS and its stability during absorption remained unknown.

In this study, the prevention and therapeutic effect of peptide OG-5 on advanced AS in ApoE^−/−^ mice was monitored. We also evaluated the bioavailability of peptide OG-5 via a Caco-2 monolayer transport experiment and metabolism experiment. In terms of mechanism, we evaluated its effect on the phenotype switch of VSMCs. This research provides a novel therapeutic approach for targeting atherogenesis through P2Y12 antagonism, and it sheds light on the potential of OG-containing peptides as a promising dietary supplement for the management of AS.

## 2. Materials and Methods

### 2.1. Materials and Chemicals

Peptide OGEFG (OG-5) (purity > 98%) was synthesized by GL Biochem Ltd. (Shanghai, China). Aspirin was obtained from Sigma-Aldrich (Shanghai, China). Fetal bovine serum (FBS) and Dulbecco’s modified Eagle medium (DMEM) were obtained from Gibco Life Technologies (Shanghai, China). IL-6 ELISA kits, penicillin streptomycin and Hank’s buffered saline solution (HBSS) were purchased from Beijing Solarbio Science and Technology Co., Ltd. (Beijing, China). TNF-α ELISA kit and cholesterol assay kit were purchased from Beyotime Biotechnology Co., Ltd. (Shanghai, China). TXB2 and soluble p-selectin ELISA kit were purchased from Nanjing Jiancheng Bioengineering Institute (Nanjing, China). Oxidized LDL (ox-LDL) was purchased from Yiyuan Biotechnology Co., Ltd. (Guangzhou, China). Primary antibody α-SMA and β-tubulin were purchased from Abcam (Shanghai, China). Other chemicals used were analytical grade or better.

### 2.2. Animal Model and Environmental Conditions

The animal study was performed in compliance with the requirements of the Guide for the Care and Use of Laboratory Animals (NIH publication No.86-23, revised 1996) and was approved by the Welfare Committee of Pony Testing International Group Co., Ltd., Beijing, China (PONY-2021-FL-12, approval date: 5 December 2021). The 7-week-old male ApoE^−/−^ mice and C57BL/6J mice were purchased from Beijing Vital River Laboratory Animal Technology Co., Ltd. (Beijing, China). The mice were acclimatized for a period of one week in a pathogen-free environment, maintained under a controlled 12-h light/dark cycle at a constant temperature of 21 °C. During this period, they had unrestricted access to a standard rodent chow diet and tap water. Following acclimatization, the mice were assigned to experimental groups randomly, with each group consisting of eight mice.

### 2.3. Treatment of ApoE^−/−^ Mice with OG-5

**Figure 1:**
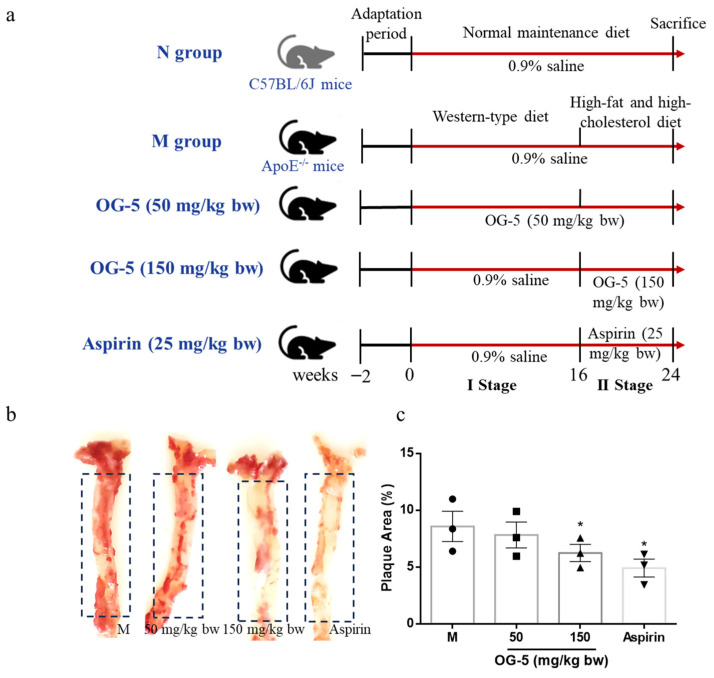
Effect of peptide OG-5 on atherosclerotic plaque formation in aorta. (**a**) Study protocol for peptide OG-5 intervention in mice. (**b**) Oil red O staining of whole aortas. (**c**) The quantification of plaque area (%) of the whole aortas in dashed box area in ApoE^−/−^ mice. Data were expressed as mean ± SEM (n = 3). Each image is representative of three mice derived from each group. * indicates indicates *p* < 0.05 as compared to the M group.

The establishment of the AS model in ApoE^−/−^ mice was divided into two stages. At I stage, 8-week-old male ApoE^−/−^ mice were fed a Western-type diet (42% fat; 21% anhydrous milk fat, 0.15% cholesterol) for 16 weeks to establish an early AS model. At II stage, a high-fat and high-cholesterol diet (containing 5% cholesterol, 0.5% sodium cholate, 20% fat) was replaced at the 17th week to accelerate the progress of AS till the 24th week. The C57BL6/J mice were utilized as the normal control group and maintained on a standard maintenance diet (20% protein; 5% fat; 50% carbohydrate; 5% cellulose; 10% vitamins and minerals). The mice were subsequently randomized into 4 groups, each consisting of 8 animals: (i) M Group (0.9% saline for ApoE^−/−^ mice), (ii) Sustainable OG-5 intervention group (50 mg/kg bw) during two stages, (iii) OG-5 treatment group (150 mg/kg bw), and (iv) aspirin group (25 mg/kg bw) at II stage. The OG-5 and aspirin were orally administered to mice every other day. The detailed information for groups is shown in Figure 1a. Animals that were not subjected to any treatment and maintained on a normal diet served as the control group (N group). The body weight was monitored every week.

After 24-week treatment, the mice were fasted overnight and sacrificed. Blood was collected to stand at room temperature for 2 h, and the serum was separated by centrifugation for further analysis.

### 2.4. ELISA Analysis

The contents of IL-6, TNF-α and p-selecin in serum were examined using commercially available ELISA kits accordingly. All experimental procedures were rigorously conducted in adherence to the manufacturer’s instructions.

### 2.5. Dissection and Examination of Aorta for Atherosclerotic Lesions

The severity of atherosclerotic lesions in the aorta was evaluated using a previous method [17]. For each experimental group, tissues comprising the myocardium, aortic root, and the entire aorta were harvested for histopathological examination. However, during the sampling of aortic root tissue, the use of ophthalmic forceps to isolate aortic root tissue may compromise the structural integrity of the myocardial tissue, leading to the collection of either myocardial tissue alone or aortic root tissue from the same mouse, but not necessarily both from the same specimen. Specifically, the entire aortas were embedded in OCT compound and preserved at −80 °C until being subjected to oil red O staining (n = 3). The aortic root, which is contiguous with the heart tissue, was utilized for H&E staining (n = 3). Additionally, the remaining tissues were collected for H&E staining of the myocardium (n = 2). Sections of the aortic root, each with a thickness of 4 μm, were continuously sliced in the cross-sectional direction and subsequently embedded in paraffin. Routine histopathological analysis was conducted using H&E staining to assess and quantify the lesion area throughout the aortic valve. For oil red O-staining, the perivascular adipose tissue was meticulously removed, and the vessels were opened longitudinally prior to undergoing oil red O staining. Images of the stained aortas were captured using a digital camera (Nikon, Tokyo, Japan). The quantification of aortic lesions was quantitatively analyzed using Image-Pro Plus software (Version 6.0, Media Cybernetics, Inc., Rockville, MD, USA).

### 2.6. VSMC Culture and Phenotype Switch

The mouse vascular smooth cell MOVAS cell line, purchased from Zhongke Quality Inspection Biotechnology Co., Ltd., Beijing, China, was incubated with DMEM supplemented with 10% FBS and 1% penicillin streptomycin at 37 °C. Ox-LDL was employed to in vitro induce VSMC phenotype switch according to a previous study [18] with some modification. Prior to peptide treatment and oxidized low-density lipoprotein (ox-LDL) stimulation, the cells were subjected to overnight serum deprivation. Subsequently, the peptide, dissolved in DMEM containing 5% FBS, was introduced to the cells. Following an 8 h treatment with the peptide, ox-LDL was added to achieve a final concentration of 50 μg/mL, and the cells were further incubated for 48 h. The supernatant and cells were collected for cholesterol determination using a commercial assay kit.

### 2.7. Western Blot

Immunoblot assays were conducted following a previously established protocol [19]. Following peptide treatment and stimulation with ox-LDL, VSMCs were lysed in RIPA buffer containing the protease inhibitor PMSF. The lysed samples were heated at 100 °C for 5 min and then separated by 10% SDS-PAGE. The resolved proteins were subsequently transferred onto a PVDF membrane. The membrane was probed with primary antibodies specific to α-smooth muscle actin (α-SMA) and β-tubulin, diluted to a ratio of 1:1000 (*v*:*v*). The visualization of antibody-bound proteins was accomplished through incubation with the respective secondary antibodies, followed by detection using enhanced chemiluminescence (ECL) with a Tanon infrared image system.

### 2.8. Peptide Transport Experiment

The Caco-2 cell culture and the establishment of the transepithelial transport model were according to the previous study [20]. The Caco-2 cells (10–20 passages) were grown in DMEM supplemented with 10% FBS and 1% penicillin streptomycin at 37 °C.

For the establishment of the transepithelial transport model, Caco-2 cells were seeded onto permeable polyester inserts (12 mm diameter, with a growth surface area of 1.12 cm^2^ and a pore size of 0.4 μm, sourced from Costar, Corning, NY, USA) placed in 12-well transwell plates at a density of 1 × 10^5^ cells per insert. Over a period of 21 days, the cell culture medium was replaced cautiously every two days to facilitate the differentiation of Caco-2 cells into fully confluent monolayers. Only monolayers exhibiting TEER values exceeding 800 Ω·cm^2^ were selected for subsequent transport experiments, ensuring the integrity and functionality of the cellular barrier [21]. The Caco-2 cell monolayers were delicately rinsed twice with HBSS and subsequently pre-incubated at 37 °C for 1 h. After pre-incubation, the HBSS in the donor chamber was exchanged with 0.5 mL of a peptide solution dissolved in HBSS, which was then introduced to the apical side. Concurrently, 1.5 mL of fresh HBSS was added to the basolateral chamber. After an incubation period of 2 h, the permeate that had traversed the monolayer and accumulated in the basolateral side was carefully collected for subsequent analysis using HPLC.

Transport percentage (%) is calculated by the following equation:

(1) $$Transport\;percentage\;\left(\%\right)=\frac{transported\;amount\;of\;peptide\;in\;basolateral\;side}{intial\;amount\;of\;peptide\;in\;apical\;side}\times 100$$

The apparent permeability coefficient (*P_app_*, cm/s) was calculated as follows:

(2) $$P_{app}=\frac{dQ}{dt}\times \frac{1}{A}\times \frac{1}{C_{0}}$$

where $\frac{dQ}{dt}$ is the permeability rate (mmol/s); *A* is the area of the membrane (cm^2^); *C*_0_ is the initial peptide concentration in the donor chamber (mM).

### 2.9. Peptide Stability in Plasma

The assessment of peptide stability in plasma was conducted with modifications to a previously described method [22]. Briefly, blood samples were collected in heparinized tubes and immediately centrifuged to separate the plasma supernatant. Subsequently, OG-5 was added to achieve a final concentration of 0.5 mM, and the mixture was incubated at 37 °C. At specified time points (0, 0.5, 1.0, 2.0, 4.0, and 8.0 h), the mixture was centrifuged using a 3 kDa ultrafiltration centrifugal tube to separate the filtrate. The filtrate was then analyzed using a Shimadzu LC-15 HPLC system, equipped with an SPD-15 ultraviolet detector (Kyoto, Japan). The HPLC analysis conditions included an injection volume of 10 μL, a flow rate of 1 mL/min, and a gradient elution program: 0–25 min, 0–25% acetonitrile (buffer B); 25–30 min, 25–35% buffer B; 30–35 min, 35–100% buffer B. The quantity of peptide OG-5 in the plasma was determined based on its peak area.

### 2.10. In Vivo Absorption Experiment on Rats

The peptide absorption in vivo was conducted as previously described [22]. The rats were orally administered with peptide OG-5 (1.0 g/kg bw). Blood samples were obtained via a minimal incision at the tail tip at 0, 0.5, 1, and 2 h after oral administration. The collected blood was transferred into 5 kDa ultrafiltration centrifugal tube, and the filtrate was collected for HPLC analysis. The elution program was conducted as follows: 0–5 min, 10–15% buffer B (acetonitrile); 5–20 min, 15–25% buffer B; 20–25 min, 25–100% buffer B.

### 2.11. Statistical Analysis

The data presented are expressed as the mean ± standard of at least three independent experiments. Statistical analysis was performed using ANOVA followed by Dunnett’s test, implemented through SPSS software (Version 19.0, IBM Inc., Armonk, NY, USA). Differences between groups were considered statistically significant when *p* < 0.05 and extremely significant when *p* < 0.01.

## 3. Results

### 3.1. Effect of OG-5 on Body Weight, Organ Index, and Serum Lipid Levels in ApoE^−/−^ Mice

The body weight and organ index could preliminarily reflect the health status and whether the sample has an obvious toxicological effect on mice. Here, we monitored the change in body weight, thymus index and spleen index of mice after oral administration of peptide OG-5 as well as aspirin. As shown in Table 1, prolonged administration of OG-5 did not elicit any adverse effects on the growth of mice, as evidenced by the absence of statistically significant differences in body weight when compared to the mice in M group. Comparable findings were noted in both the thymus index and spleen index between the OG-5-treated group and the M group.

The level of lipid in serum is an important index that reflects the lipid metabolism. Compared to the N group, the M group exhibited elevated levels of total cholesterol (TC), triglycerides (TGs), and LDL-C, while HDL-C levels were significantly decreased (*p* < 0.05). Eight-week high-fat and high-cholesterol diet resulted in further elevation of serum lipid levels (M-II stage versus M-I stage), indicating a deeper severity of abnormal lipid metabolism. The treatment of peptide OG-5 had a limited effect on the level of serum lipids, though only sustained administration of a low dose of OG-5 attenuated the content of TC and LDL-C. The above results suggest that peptide OG-5 may inhibit AS via another mechanism rather than the regulation of abnormal lipid metabolism.

### 3.2. Peptide OG-5 Attenuated Atherogenesis in ApoE^−/−^ Mice

Our previous study has shown that peptide OG-5 has a prevention effect on early progression of AS [12]. However, its prevention effect on middle- or late-period AS and whether peptide OG-5 had a therapeutic effect on AS remained unknown. To further evaluate the effect of peptide OG-5 on atherogenesis in vivo, ApoE^−/−^ mice were subjected to a dietary regimen rich in both fat and cholesterol, aimed at accelerating the development of AS after 16-week modeling (Figure 1a). The oil red O staining results depicted in Figure 1b illustrate the differential impact of peptide OG-5 on atherosclerotic lesion formation in the aortas of mice. Notably, the M group mice exhibited a substantial number of atherosclerotic lesions spanning the entire aorta, whereas the mice treated with peptide OG-5 displayed significantly smaller and fewer atherosclerotic lesions, highlighting the protective effect of OG-5 against the development of AS. Treatment with peptide OG-5 at a dose of 150 mg/kg bw significantly reduced the aortic plaque formation area by 30% (*p* < 0.05), an effect comparable to that of aspirin (25 mg/kg bw). Meanwhile, a lower bleeding risk was observed after long-term oral administration of peptide OG-5 at a dosage of 150 mg/kg bw compared to that of aspirin (Figure S1, Supporting Information).

**Figure 2:**
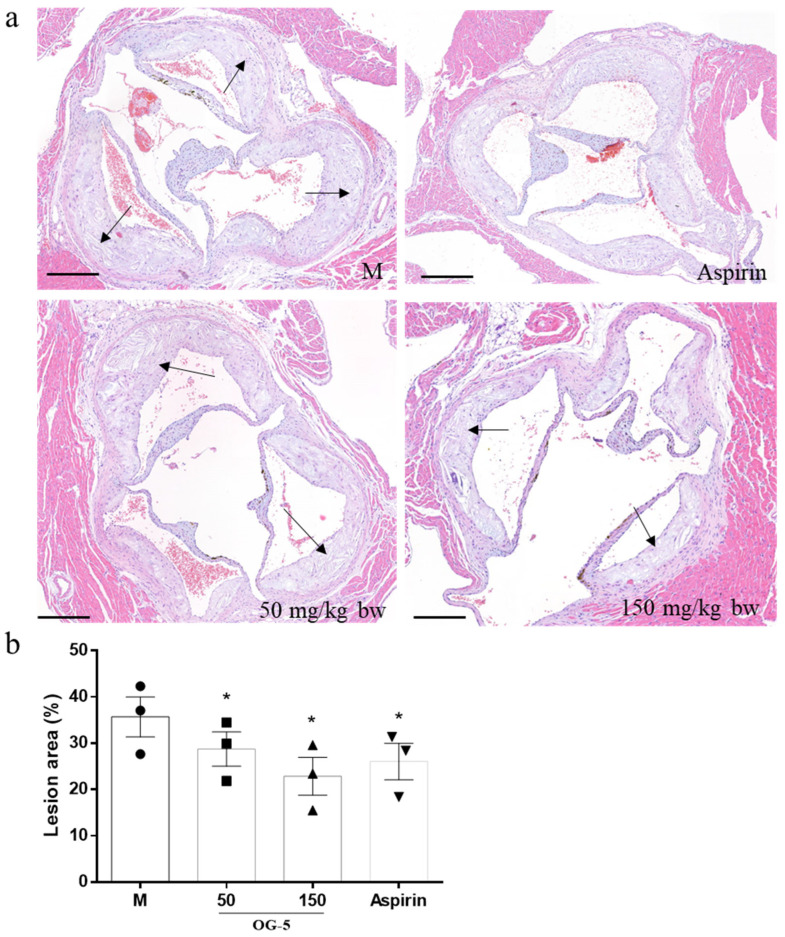
Comparison of the development of atherosclerotic plaques in aortic root of ApoE^−/−^ mice treated with different concentrations of OG-5, versus those treated with saline. (**a**) Representative images of H&E staining of the aortic root sections. (**b**) Quantification of the lesion areas in aortic root sections. Data are expressed as mean ± SEM. Each image is representative of three mice derived from each group. * indicates indicates *p* < 0.05 as compared to the M group. The arrow indicates atherosclerotic plaques.

The H&E staining of the proximal aorta (aorta root sections) is shown in Figure 2. The proximal aorta in M group mice had obvious intimal hyperplasia and fatty plaques, and a large number of foam cells were accumulated in the plaques. Oral administration of peptide OG-5 reduced fatty plaque accumulation and inhibited necrotic core area formation at a dose of 50 mg/kg bw. The therapeutic effect under 150 mg/kg bw was better than the low-dose intervention of peptide OG-5 starting from the modeling stage I (19.3% vs. 35.9%, *p* < 0.05). Notably, while the low-dose intervention of peptide OG-5 during the initial modeling stage (Stage I) showed only a modest ability to inhibit plaque formation throughout the aorta (Figure 1c), sustained treatment with peptide OG-5 at a dose of 50 mg/kg bw significantly improved the condition of atherosclerotic plaques in the aortic root (*p* < 0.05) (Figure 2). Importantly, the aortic root is recognized as the primary site where fatty deposits accumulate during the development of AS. These results indicate that continuous treatment with OG-5 may help delay the progression of AS, particularly in its early to middle stages.

**Figure 3:**
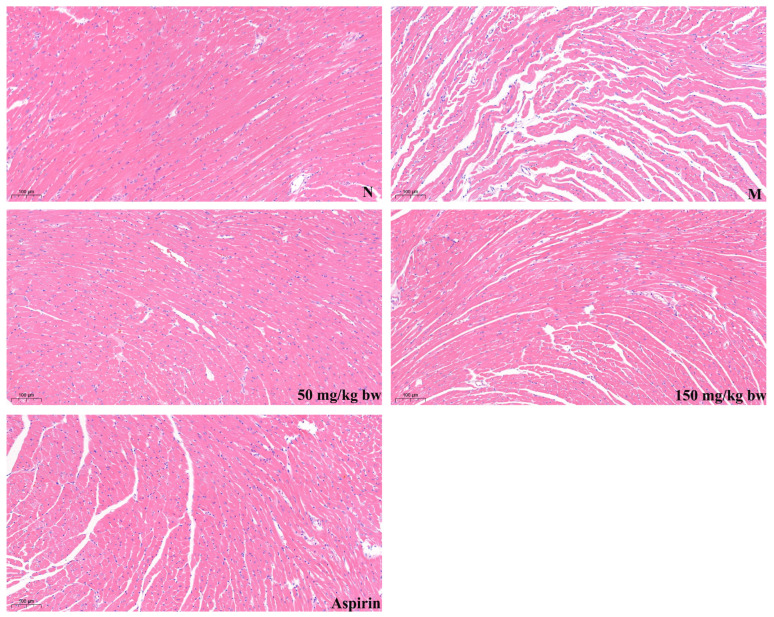
Comparison of the myocardium tissue in ApoE^−/−^ mice treated with different concentrations of OG-5, versus those treated with saline. Representative images of H&E staining of the myocardium tissue. Each image is representative of two mice derived from each group. The arrow indicates atherosclerotic plaques.

The H&E staining results of mouse myocardial tissue are shown in Figure 3. The myocardial cells of N group mice were arranged in a complete manner with clear nuclei, while the myocardial cells of M group mice were arranged in a disordered manner, with obvious cell fiber proliferation, and a small amount of cell nuclei were shed, indicating the presence of necrotic myocardial cells. The peptide OG-5 intervention group exhibited a significant improvement for the myocardial cell morphology compared to the model group. Moreover, the effect of ingesting peptide OG-5 (50 mg/kg bw) in the modeling stage I was better than the therapeutic intervention of peptide OG-5 (150 mg/kg bw) and aspirin (25 mg/kg bw) in the modeling stage II.

### 3.3. Peptide OG-5 Inhibited Inflammatory Cytokines and Platelet Activation in Plasma

**Figure 4:**
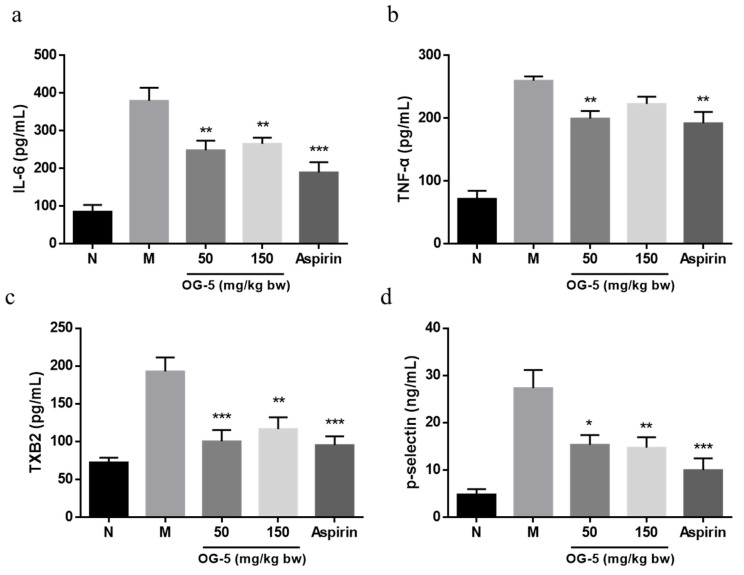
Peptide OG-5 inhibited pro-inflammatory cytokine release and platelet activation in ApoE^−/−^ mice. The plasma concentrations of (**a**) IL-6, (**b**) TNF-α, (**c**) TXB2, and (**d**) p-selectin were determined using commercial ELISA kits following 16 weeks of oral administration of OG-5 (n = 8). Statistical significance was indicated by * (*p* < 0.05) , ** (*p* < 0.01) and *** (*p* < 0.001) when compared to the M group.

The results of the contents of platelet activation factors and inflammatory cytokines in plasma of mice are shown in Figure 4. The content of IL-6, TNF-α, TXB2 and p-selectin in M group mice was significantly increased compared with that of N group mice. This result indicates that the platelets in ApoE^−/−^ mice became more susceptible to agonists when compared with normal mice. Oral administration of peptide OG-5 significantly attenuated the secretion of IL-6, TNF-α as well as the level of TXB2 and p-selectin content in plasma. Furthermore, the effect of peptide OG-5 in the sustainable intervention group (50 mg/kg bw) was better than that in the treatment group (150 mg/kg bw), which was comparable to the effect of aspirin (25 mg/kg bw) in the positive control group.

### 3.4. Peptide OG-5 Inhibited VSMC Phenotype Switch Induced by Ox-LDL

**Figure 5:**
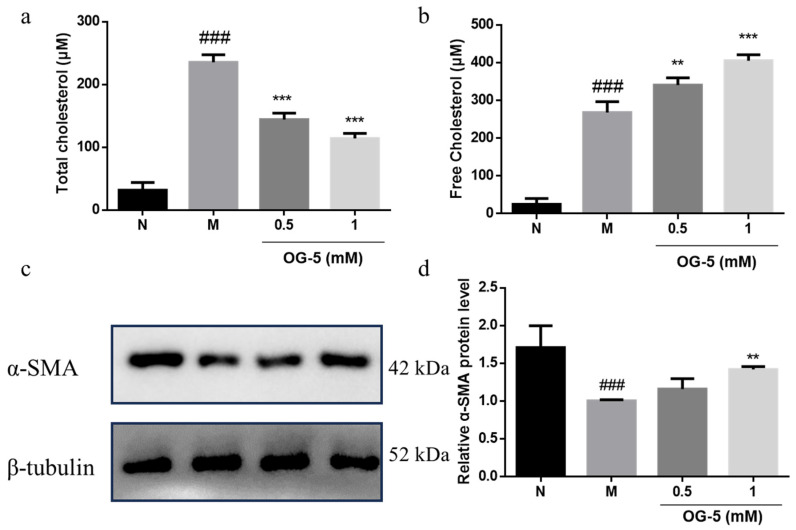
Peptide OG-5 inhibited phenotype switch of vascular smooth muscle cell (VSMC) from contractile cell type stimulated by ox-LDL. The content of (**a**) the total cholesterol in VSMCs and (**b**) the free cholesterol in supernatant (n = 3). (**c**) Immunoblot analysis of VSMCs with specific antibody against α-SMA and β-tubulin. Quantification of protein (**d**) α-SMA was performed by densitometric analysis of the immunoblots. ** and *** indicates *p* < 0.01 and *p* < 0.001, respectively, as compared to the M group. ### indicates *p* < 0.001 as compared to the N group.

VSMCs are the major cell type in AS-prone arteries and take up excess lipids after the phenotype switch to macrophage-like cells, thereby contributing to luminal occlusion [23]. To further investigate the regulation mechanism of peptide OG-5 on AS, we evaluated its effect on the phenotype switch of VSMCs in vitro. As shown in Figure 5, peptide OG-5 inhibited the uptake of cholesterol in VSMCs and promoted the release of free cholesterol to the supernatant in a dose-dependent manner (Figure 5a,b). VSMCs stimulated with Ox-LDL (M group), representing the synthetic phenotype, exhibited decreased expression levels of the contractile phenotype marker α-SMA compared to the N group. Treatment with the peptide OG-5 at a dosage of 1 mM significantly attenuated the changes induced by Ox-LDL (*p* < 0.01) (Figure 5c,d). These results indicate that OG-5 could reduce the accumulation of lipoprotein in VSMCs and inhibit the phenotype switch of VSMCs.

### 3.5. Transport of OG-5 Across Caco-2 Cell Monolayers

**Figure 6:**
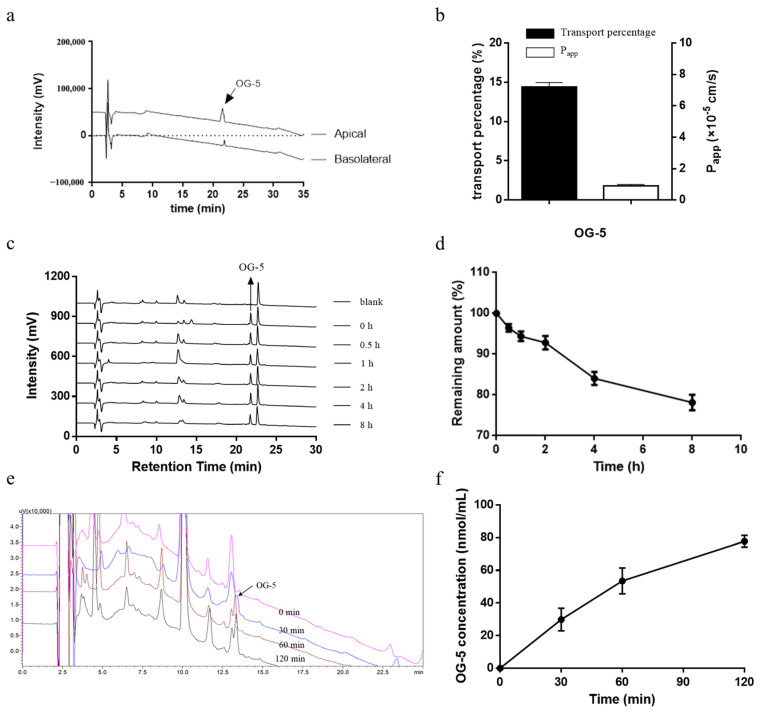
Bioavailability of peptide OG-5. (**a**,**b**) Transport of OG-5 across Caco-2 cell monolayers. (**a**) HPLC profile of OG-5 in apical and basolateral side and (**b**) its transport ratio and P_app_ value. (**c**,**d**) Stability of peptide OG-5 in rat plasma. (**c**) HPLC profile of peptide OG-5 in rat plasma and (**d**) its remaining amount with different incubation time. (**e**,**f**) Stability of peptide OG-5 after oral administration. (**e**) HPLC profile of peptide OG-5 in rat plasma after oral administration and (**f**) its concentration with different time.

Based on the above observation that peptide OG-5 could reduce the accumulation of lipoprotein in VSMCs and inhibit the phenotype switch of VSMCs to attenuate AS, we further evaluated the bioavailability and stability of peptide OG-5 during absorption. The Caco-2 cell line has been widely used to simulate intestinal absorption in vitro [24]. Transepithelial transport of peptide OG-5 was carried out, and HPLC analysis was employed to analyze the content in each side. As shown in Figure 6a, peptide OG-5 was transported across the Caco-2 cell monolayers intact with a retention time of 21.4 min, indicating that peptide OG-5 could resist peptidase degradation.

The P_app_ values of peptide OG-5 are shown in Figure 6b. The transport percentage of OG-5 was 14.40% ± 0.57, which is much higher than those of casein peptides VPP and IPP and ovotransferrin-derived peptide IRW, as described previously [25,26]. Similarly, peptide OG-5 also exhibited a high P_app_ value with 1.80 × 10^−5^ cm/s. The P_app_ value of peptide OG-5 was much higher than the results reported for other peptides such as LY (0.35 × 10^−5^ cm/s), RVPSL (0.70 × 10^−5^ cm/s) and TNGIIR (0.49 × 10^−5^ cm/s) [20,27,28]. According to the previous study, a P_app_ value higher than 1.0 × 10^−5^ cm/s suggests that the compound can be absorbed well (70–100%) in vivo [29]. These results demonstrated that OG-5 can be absorbed well in humans.

### 3.6. Stability of Peptide In Vitro Plasma

The application of peptides is usually limited for the short half-life and is commonly due to fast renal clearance as well as enzymatic degradation, including aminopeptidases, carboxypeptidases, dipeptidases and endopeptidases occurring during systemic circulation [30,31]. The stability of peptide OG-5 in plasma was evaluated, and the result is presented in Figure 6c,d. Notably, after an 8-h incubation period at 37 °C, 78.2% of the peptide remained intact in plasma, suggesting that peptide OG-5 exhibits a high degree of stability within the blood environment. This result is consistent with the peptide OG and OGE with a remaining amount >70% after 8 h of incubation as described in our previous study [22]. Interestingly, the half-life of pentapeptide OG-5 was longer than that of PGEOG (4.3 h) [22]. This might be owing to the hydroxylation of Pro in the *N*-terminal. The present study suggests that peptide OG-5 had good stability in plasma to exert its biological activity in vivo.

### 3.7. In Vivo Absorption of Peptide OG-5

To further evaluate the absorption of peptide OG-5, an in vivo absorption experiment was performed. As shown in Figure 6d–f, the plasma concentration of peptide OG-5 increased significantly after oral administration for 30 min. The peak concentration was observed at 120 min with 77.8 nmol/mL, demonstrating its robust stability during in vivo gastrointestinal digestion and absorption. This finding suggests that peptide OG-5 is capable of remaining intact in the bloodstream, underscoring its potential for effective delivery and therapeutic action.

## 4. Discussion

Collagen is widely distributed in fishery by-products and is a natural source of peptides. In our prior study, OG-containing peptides were successfully isolated and characterized from CHs derived from *Salmo salar* and *Hypophthalmichthys molitrix*, demonstrating potent antiplatelet aggregation activity, particularly against ADP-induced aggregation [11,22]. Among these peptides, OG-5 emerged as one of the most effective in inhibiting platelet aggregation. Furthermore, OG-5 also exhibited inhibitory effects on thrombus formation in both a FeCl_3_-induced arterial thrombosis model and a carrageenan-induced mouse tail thrombosis model [11]. Our previous in vivo study showed that oral administration of both peptide OG-5 and CHs from *Salmo salar* could prevent the formation of AS in ApoE^−/−^ mice caused by a fat diet [12,32], indicating its potential for the primary prevention of AS. However, its prevention effect on the middle or late period of AS and whether peptide OG-5 had a therapeutic effect on AS remained unknown.

This study constitutes a sequential experiment building upon the findings of prior animal experimentation. Initially, we assigned 18 mice to each group. Over the course of the 16-week experiment, we noted that collagen peptide OG-5 demonstrated a favorable prophylactic effect against early-stage atherosclerosis [12]. To delve deeper into its in vivo efficacy, we proceeded to administer a high-fat and high-cholesterol diet to expedite the atherosclerotic process. Consequently, due to the necessity of sacrificing some mice for the preceding experiment, only 8 mice per group were retained for the current investigation.

The low-dose sustainable intervention of peptide OG-5 from the stage I of modeling improved the condition of atherosclerotic plaques in the aortic root without bleeding risks. Notably, the myocardial cell morphology was also ameliorated significantly. The embolism caused by AS of the aortic valve is related to the formation of coronary heart disease, suggesting that OG-5 peptide may also have the potential to prevent coronary heart disease at a dose of 50 mg/kg bw. The inhibitory effect of peptide OG-5 under 150 mg/kg bw was better than the low-dose sustainable intervention in the middle and late period of AS. However, a little bleeding risk was observed after oral long-term administration of peptide OG-5 at a dosage of 150 mg/kg bw when compared with that of the M model (Figure S1). Although the bleeding time is prolonged at a dose of 150 mg/kg bw, it is still shorter compared to aspirin, resulting in a lower risk of bleeding. These results indicate that collagen peptide is more suitable for early intervention of AS. Furthermore, we also investigated the bioavailability of peptide OG-5 via the Caco-2 monolayer transport experiment and metabolism experiment in rats. Mice and rats are both widely used in in vivo drug metabolism experiments. In this study, we used rats to perform this experiment. The main reason is that the individual differences in rats are relatively small compared to mice, and the reproducibility and reliability of experimental results are higher. The results showed that peptide OG-5 could resist gastrointestinal digestion in vivo as well as enzymatic degradation and exhibit good stability in plasma.

The transition of foam cells into plaques is a crucial step in the initiation and progression of AS. VSMCs play a pivotal role in this process, as they are the primary cell type involved in early arterial intimal thickening and also significantly contribute to the formation of foam cells [33,34]. The mechanism for the generation of foam cells involves the phenotype switch of VSMCs from contractile differentiated cells to synthetic phenotype, which is associated with reduced expression of contractile markers and increased expression of synthetic markers. The accumulation of excess lipids within VSMCs acts as a catalyst for foam cell formation, thereby contributing to the overall intimal foam cell population found in atherosclerotic lesions. Additionally, research has revealed that the activation of the P2Y12 receptor has detrimental effects, as it reduces cholesterol efflux and fosters the development of VSMC-derived foam cells [16]. Numerous instances of both clinical and experimental evidence suggest that the P_2_Y_12_ receptor may not only amplify and maintain ADP-induced platelet aggregation, but also play a role in atherogenesis by facilitating the proliferation and migration of VSMCs [13,35,36,37,38]. Our previous study showed that the peptide OG-5 could inhibit platelet aggregation induced by ADP, via antagonism effects on P_2_Y_12_ receptors to regulate the secretion of platelet granules [11,12]. In this study, we detected the contractile protein markers’ expression in VSMCs and found that peptide OG-5 at high doses (1 mM) could reduce the accumulation of lipoprotein in VSMCs and attenuate the phenotype switch of VSMCs induced by ox-LDL. Based on the above results, peptide OG-5 has multifunctional effects on platelet activation as well as VSMC phenotype switch to regulate AS progression, which also supports the idea that the P_2_Y_12_ receptor inhibitor may also serve as an effective agent for anti-atherogenesis intervention. With future chemical modifications, encapsulation, and other methods, there is potential to further enhance its activity, indicating its potential application value in the treatment of AS.

## 5. Conclusions

In summary, we investigated the effect of peptide OG-5 on advanced atherosclerotic lesions in ApoE^−/−^ mice and its stability during absorption. The results provide evidence that oral administration of OG-5 at 150 mg/kg bw reduced the aortic plaque formation area by 30% in ApoE^−/−^ mice with a reduced bleeding risk. Intervention with a low dose of OG-5 (50 mg/kg bw), initiated in the early stage of atherosclerosis, continues to provide benefits in the middle and late stages without bleeding risks. Furthermore, peptide OG-5 had good stability in plasma and reached maximum concentration in blood after 120 min. These findings reveal that peptide OG-5 has the potential to serve as an effective agent for anti-atherogenesis intervention.

## Figures and Tables

**Table 1:** Effect of oral administration of peptide OG-5 on body weight, organ index, and serum lipid levels in ApoE^−/−^ mice (n = 8).

Parameters	N	M-I-Stage	M-II-Stage	OG-5 (50 mg/kg bw)	OG-5 (150 mg/kg bw)	Aspirin
Initial Body weight (g)	24.0 ± 1.3	24.9 ± 0.9	27.4 ± 2.5	24.5 ± 1.0	27.8 ± 2.5	27.7 ± 2.8
Final Body weight (g)	36.6 ± 3.3	27.4 ± 3.0 ^#^	28.2 ± 1.4	27.7 ± 2.2	28.9 ± 2.0	28.8 ± 1.7
Thymus index (mg/g bw)	0.78 ± 0.09	0.81 ± 0.24	0.83 ± 0.24	0.69 ± 0.15	0.79 ± 0.13	0.83 ± 0.12
Spleen index (mg/g bw)	3.05 ± 0.52	6.60 ± 2.91 ^#^	5.01 ± 1.98	4.16 ± 0.99	4.80 ± 2.13	4.99 ± 2.64
TC (mM)	5.21 ± 0.88	35.16 ± 3.74 ^#^	45.52 ± 3.79 ^†^	36.48 ± 4.91 **	40.99 ± 5.78	41.09 ± 6.05
TG (mM)	0.44 ± 0.09	1.56 ± 0.02 ^#^	3.29 ± 0.42 ^†^	3.04 ± 0.31	3.25 ± 0.78	3.43 ± 0.96
HDL-C (mM)	2.10 ± 0.52	0.73 ± 0.12 ^#^	0.56 ± 0.10	0.66 ± 0.10	0.61 ± 0.27	0.64 ± 0.37
LDL-C (mM)	0.31 ± 0.12	6.81 ± 0.98 ^#^	10.28 ± 0.79 ^†^	8.31 ± 0.71 *	9.39 ± 2.03	9.55 ± 1.89

Notes: ^#^ indicates *p* < 0.05 as compared to the N group. ^†^ indicates *p* < 0.05 as compared to the M-I-Stage group. * and ** indicate *p* < 0.05 and *p* < 0.01, respectively, as compared to the M-II-Stage group.

## Data Availability

The data presented in this study are available on request from the corresponding author. The data are not publicly available due to privacy.

## Supplementary Materials

The following supporting information can be downloaded at: https://www.mdpi.com/article/10.3390/nu16213752/s1, Figure S1: Bleeding time after oral administration of peptide OG-5 in ApoE^−/−^ mice (n = 8).
