# Supplementary material for: Effect of Oral Administration of Collagen Peptide OG-5 on Advanced Atherosclerosis Development in ApoE−/− Mice
Source: Nutrients. 2024 Oct 31;16(21):3752. doi: 10.3390/nu16213752 (PMC11547735; doi:10.3390/nu16213752)
Supplement: Supplementary file 1 [file nutrients-16-03752-s001.zip › nutrients-3254156-supplementary.pdf]

Supplementary figure legend:

Figure S1. Bleeding time after oral administration of peptide OG-5 in ApoE$^{-/-}$ mice ($n = 8$). Statistical significance was indicated by ** ($p < 0.01$) and *** ($p < 0.001$) when compared to the M group.

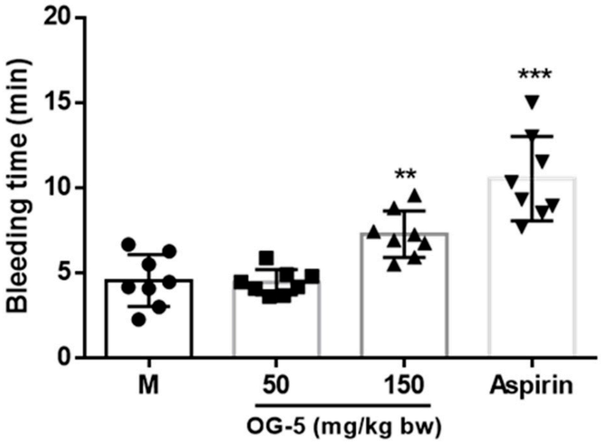

Figure S1.
